# Soil Phosphorus Availability Modulates Host Selectivity of *Pedicularis kansuensis* Between Legumes and Grasses

**DOI:** 10.3390/plants14152356

**Published:** 2025-07-31

**Authors:** Xiaolin Sui, Ruijuan Xue, Airong Li

**Affiliations:** 1 Yunnan Key Laboratory for Wild Plant Resources, Department of Economic Plants and Biotechnology, Kunming Institute of Botany, Chinese Academy of Sciences, Kunming 650201, China; suixiaolin@mail.kib.ac.cn (X.S.);; 2 Key Laboratory of Phytochemistry and Natural Medicines, Kunming Institute of Botany, Chinese Academy of Sciences, Kunming 650201, China

**Keywords:** host selectivity, legume–grass mixture, parasitic plant, phosphorous availability, haustorium, *Pedicularis kansuensis*

## Abstract

Host selectivity or preference plays a critical role in enabling parasitic plants to identify suitable hosts and influence plant community dynamics. Phosphorus (P) is known to affect the growth of root hemiparasitic plants and their interaction with single host species, but its role in shaping host selectivity across multiple hosts is unclear. In a pot experiment, we used a grass–legume co-culture design and evaluated whether the root hemiparasitic plant *Pedicularis kansuensis* exhibits selective parasitism on legumes (*Medicago sativa*) versus grasses (*Elymus nutans*) and assessed the impact of soil P availability on this preference. The results showed that *P. kansuensis* inhibited the growth of both host species, but the magnitude of suppression varied with P availability. Under low P conditions, *P. kansuensis* preferentially parasitized the tender *M. sativa*, causing a greater biomass reduction in the legume. In contrast, at high P levels, *P. kansuensis* decreased its foraging on legumes, shifting its parasitism towards the dominant *E. nutans*, which potentially led to stronger suppression of grass growth. Our findings demonstrate that soil P availability modulates host selectivity in *P. kansuensis*, emphasizing the influence of soil nutrient conditions on parasite–host dynamics. This research provides insights into managing the impacts of parasitic plants on plant community structure through nutrient interventions.

## 1. Introduction

Root hemiparasitic plants account for more than half of all parasitic plant species and are commonly found in diverse natural and semi-natural environments [1]. These plants extract essential resources from host plants, including carbon (C), nitrogen (N), phosphorus (P), and water, via specialized structures called haustoria [2,3]. This parasitism significantly inhibits host growth and reduces overall plant productivity [4,5]. Additionally, root hemiparasites influence interactions between host and non-host plants, altering plant community composition and diversity [6,7]. While most root hemiparasitic plants are generalists capable of parasitizing a wide range of neighboring plant species, they exhibit clear preferences for certain hosts [5]. Foraging for preferred hosts (host selectivity) is crucial for successful parasitism and has profound implications for plant community dynamics. However, the mechanisms driving host selectivity in root hemiparasitic plants remain poorly understood.

Host selectivity in root hemiparasitic plants generally occurs in three stages: growth toward the host root (host tropism), attachment to the host root, and haustorium formation. Although research into these processes is still emerging, there is evidence that root hemiparasites exhibit host tropism. For instance, a recent study demonstrated that seedlings of the facultative root hemiparasite *Rhinanthus minus* preferentially grew toward legume roots rather than grass roots in an agar plate experiment [8]. However, while this short-term (3-week) study provided direct evidence of early-stage host selectivity, it did not examine whether such tropism results in the formation of more haustoria on legume hosts or leads to greater suppression of legume biomass compared to grasses in multi-species systems. A grass–legume co-culture pot experiment found that the obligate root hemiparasite *Melampyrum arense* had a more pronounced negative impact on the legume host *Medicago sativa* than on the grass *Lolium perenne* [9]. These findings suggest that root hemiparasites may preferentially target legumes over grasses, causing uneven effects between host species. However, as obligate root hemiparasites depend entirely on their hosts for survival from germination onward [2,10], host selectivity is more critical for their survival than for facultative hemiparasites. In contrast, facultative hemiparasites can grow independently for a portion of their lifecycle and may not exhibit the same degree of host specificity [2,3]. Whether facultative hemiparasites demonstrate host selectivity and impose unequal effects on coexisting host species remains largely unexplored.

Although root hemiparasitic plants have shown a preference for suppressing legume hosts over grass hosts in the aforementioned pot experiments, this phenomenon was not consistently observed in field experiments. For example, *Rhinanthus* species typically harmed grass biomass but had varying effects on legume biomass—positive [11,12], negative [13,14], or neutral [15]. Several reasons have been proposed to explain these contrasting effects of parasitism on legumes. Firstly, different plant species have been found to differ in their quality as hosts [16], so the species diversity of legumes in field conditions may be a factor. Secondly, the abundance of legumes in the plant community also plays a role in determining the parasite’s effect on legume biomass, as the most abundant host species are more likely to be encountered by a parasite than less abundant ones [17]. Additionally, environmental factors such as nutrient availability may significantly influence host selectivity for legumes in natural habitats.

Nitrogen (N) and phosphorus (P) are essential macronutrients that influence the growth of hemiparasitic plants, and meanwhile, the primary mineral nutrients the parasites extract from their hosts [18,19]. Previous studies found that soil N and P levels impacted the growth of hemiparasitic plants [20] and their interactions with host plants [21,22,23]. However, they were mainly conducted in single host–parasite pairs, limiting our understanding of how nutrients influence the interactions between hemiparasitic plants and their coexisting hosts in natural environments. While N has been a primary focus of studies examining nutrient effects on parasitic plants [24], the role of P remains underexplored [22]. Phosphorus not only affects the growth of hemiparasitic plants [25,26] but also influences grass–legume interactions [27,28] and the secretion of plant exudates, such as flavonoids and strigolactones [29,30,31], which may act as chemical signals for host tropism [8,32]. Therefore, we hypothesize that the host selectivity of root hemiparasitic plants between grass and legume hosts may be modulated by soil P levels.

*Pedicularis kansuensis*, a facultative root hemiparasitic plant, is widespread in subalpine regions of western China [33], including the Qinghai–Tibet Plateau and Tianshan Mountains [34]. This species has a significant ecological and economic impact, as it can reduce grassland productivity by up to 80%, thereby threatening the local livestock industry [35]. Previous research indicates that *P. kansuensis* parasitizes a wide range of hosts, with a preference for species in the Poaceae (grasses) and Fabaceae (legumes) [36]. However, the host selectivity of *P. kansuensis* between coexisting grass and legume hosts and the factors that influence its host selectivity in mixed-species systems remain poorly understood. This study investigates whether *P. kansuensis* selectively parasitizes a legume host (*M. sativa*) over a grass host (*Elymus nutans*) in a grass–legume co-culture pot experiment and whether soil P availability affects this selectivity. By clarifying the interactions between *P. kansuensis* and its hosts under varying P levels, this research aims to improve our understanding of how P availability modulates parasitic preference between phylogenetically distinct hosts and to provide insights for managing the impacts of parasitic plants in grassland ecosystems.

## 2. Results

### 2.1. Haustorium Formation of the Hemiparasite

**Figure 1:**
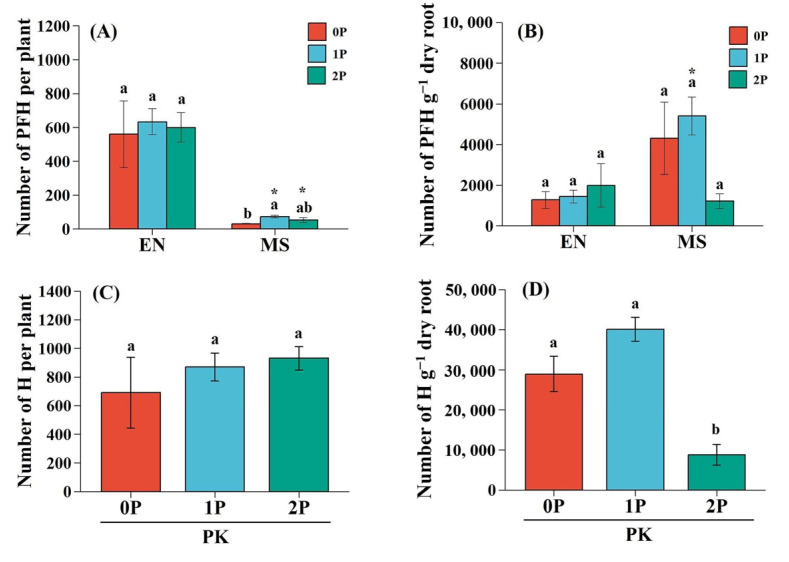
Haustoria formation capability of *Pedicularis kansuensis* under various P levels. (**A**) Number of presumably functional haustoria (PFH) produced by *P. kansuensis* on EN and MS; (**B**) number of PFH per gram dry root of EN and MS; (**C**) total number of haustoria (H) formed by PK (sum of functional/non-functional haustoria on both hosts); and (**D**) number of H per gram dry root of PK (haustoria incidence). Data are presented as mean ± 1SE. Different letters indicate statistically significant differences among P availability at a *p* < 0.05 level. Asterisks denote significant differences between EN and MS under the same P level (* *p* < 0.05). Species: *Elymus nutans* (EN), *Medicago sativa* (MS), *P. kansuensis* (PK). P treatments: 0P, 0 mM/L Na_2_HPO_4_; 1P, 1.33 mM/L Na_2_HPO_4_; 2P, 2.66 mM/L Na_2_HPO_4_.

*Pedicularis kansuensis* formed more PFH on the grass host compared to the legume host, especially under 1P (*p* < 0.05) and 2P (*p* < 0.05) treatments (Figure 1A). However, the number of PFH per gram of dry root was higher on legume roots than grass roots under low P conditions (particularly in 1P treatment, *p* < 0.05) but not under 2P conditions (*p* > 0.05, Figure 1B). P availability had no significant impact on the number of PFH on grass roots (*p* > 0.05) but did affect that in the legume host (*p* < 0.05) (Figure 1A). The total number of haustoria produced by *P. kansuensis* was not significantly affected by P availability (*p* > 0.05; Figure 1C). Conversely, the total number of haustoria per gram dry root of *P. kansuensis* was significantly decreased under 2P treatments compared to 0P (*p* < 0.01) and 1P (*p* < 0.01) conditions (Figure 1D).

### 2.2. Growth Performance and Biomass Allocation of Hosts and the Hemiparasite

**Figure 2:**
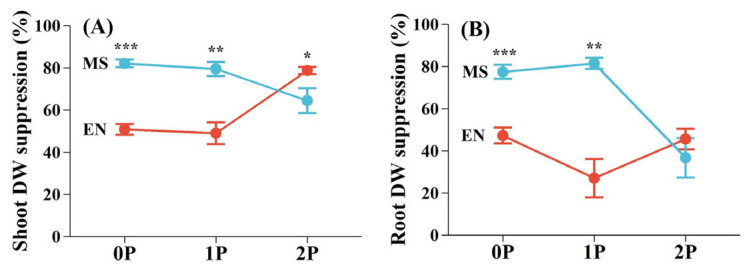
The degree of suppression of host growth by *Pedicularis kansuensis* under different P levels. (**A**) The suppression of shoot dry weight (DW); (**B**) the suppression of root DW. Data are mean ± 1 SE. Asterisks denote significant differences between species under the same P level (* *p* < 0.05; ** *p* < 0.01; *** *p* < 0.001). Species: *Elymus nutans* (EN), *Medicago sativa* (MS). P treatments: 0P, 0 mM/L Na_2_HPO_4_; 1P, 1.33 mM/L Na_2_HPO_4_; 2P, 2.66 mM/L Na_2_HPO_4_.

The significant interaction effects between P availability and parasitism on the growth performance of host plants were only found on the root DW of *M. sativa* (F = 6.08, *p* < 0.01), as the damage induced by *P. kansuensis* decreased with P increment (Table 1). Parasitism exerted a strong negative effect on both hosts, while the effect of P availability was host- and tissue-specific (Table 1). The degree of growth suppression caused by *P. kansuensis* varied between *E. nutans* and *M. sativa*, which was influenced by P availability (Figure 2). At low P levels, *M. sativa* experienced significantly greater suppression of shoot (0P, 82.08%; 1P, 79.39%) and root DWs (0P, 77.39%; 1P, 81.38%) compared to *E. nutans* (0P, shoot 50.81%, root 47.20%; 1P, shoot 48.98%, root 27.02%). However, under high P conditions (2P), growth suppression of *E. nutans* increased substantially (shoot, 78.68%; root, 45.54%), while *M. sativa* (shoot, 64.41%; root, 36.65%) showed reduced inhibition (Figure 2).

P availability had no obvious effect on the root–shoot ratio of infected (*p* > 0.05) or uninfected (*p* > 0.05) *E. nutans*, despite the root–shoot ratio of infected *E. nutans* appeared to increase with higher levels of P (Table 1). The root–shoot ratios of uninfected *M. sativa* varied significantly among P treatments, while there were no apparent differences for infected *M. sativa* (*p* > 0.05; Table 1). Compared to uninfected controls, the root–shoot ratio of infected *M. sativa* (*p* < 0.05) and *E. nutans* (*p* < 0.05) was significantly higher under 2P conditions (Table 1).

For *P. kansuensis*, its shoot DW (*p* < 0.05), root DW (*p* < 0.01), and root–shoot ratio (*p* < 0.05) were significantly affected by P availability. Higher P availability (2P) significantly increased both shoot and root DWs compared to 1P, while P deficiency (0P) did not suppress its growth (Table 1). In addition, the root–shoot ratio of *P. kansuensis* was higher under 2P than other P levels (Table 1).

### 2.3. Biomass Proportions of Host and Hemiparasite per Pot

**Figure 3:**
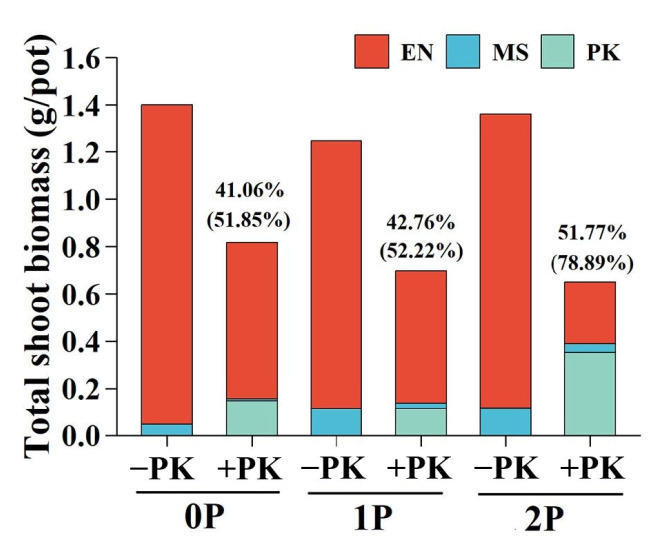
Total shoot biomass in each pot with or without *Pedicularis kansuensis* under different P levels. Data are presented as means of replicate pots under different P and parasitism conditions, with stacked bars showing the mean values of *Elymus nutans* (EN), *Medicago sativa* (MS), and *P. kansuensis* (PK) separately. In addition, the percentage of total shoot biomass per pot (including PK, outside bracket) and the percentage of total shoot biomass of both host plants suppressed by PK (inside bracket) are displayed for each P treatment. Parasitism: −PK, without PK; +PK, with PK. P treatments: 0P, 0 mM/L Na_2_HPO_4_; 1P, 1.33 mM/L Na_2_HPO_4_; 2P, 2.66 mM/L Na_2_HPO_4_.

The shoot DW proportion of *E. nutans* consistently exceeded that of *M. sativa* across all treatments (Figure 3). Infection by *P. kansuensis* reduced the shoot DW proportions of *E. nutans*, which was more serious with 2P treatment (54.63%, *p* < 0.05) than in the 0P (16.98%, *p* < 0.05) and 1P (12.41%, *p* < 0.05) conditions. Conversely, the suppression of shoot DW proportion of *M. sativa* by *P. kansuensis* was lower at high P level (2P, 30.86%, *p* > 0.05) than at low P treatments (0P, 69.39%, *p* < 0.01; 1P, 66.00%, *p* < 0.01). Infection by *P. kansuensis* significantly suppressed the root DW proportion of *E. nutans* under 2P treatment (27.80%, *p* < 0.05) but obviously inhibited that of *M. sativa* under 0P (59.78%, *p* < 0.05) and 1P (73.50%, *p* < 0.05) treatments. The shoot and root DW proportion of *P. kansuensis* was highest under 2P conditions (shoot, 52.52%; root, 25.93%) and similar between 0P (shoot, 18.72%; root, 5.23%) and 1P (shoot, 17.53%; root, 4.11%) treatments. Total shoot biomass per pot decreased significantly in the presence of *P. kansuensis* (F = 83.74, *p* < 0.001), and the percentage suppression of total shoot biomass by *P. kansuensis* increased with P availability (Figure 3).

### 2.4. Shoot N and P Status of Hosts and Hemiparasite

**Figure 4:**
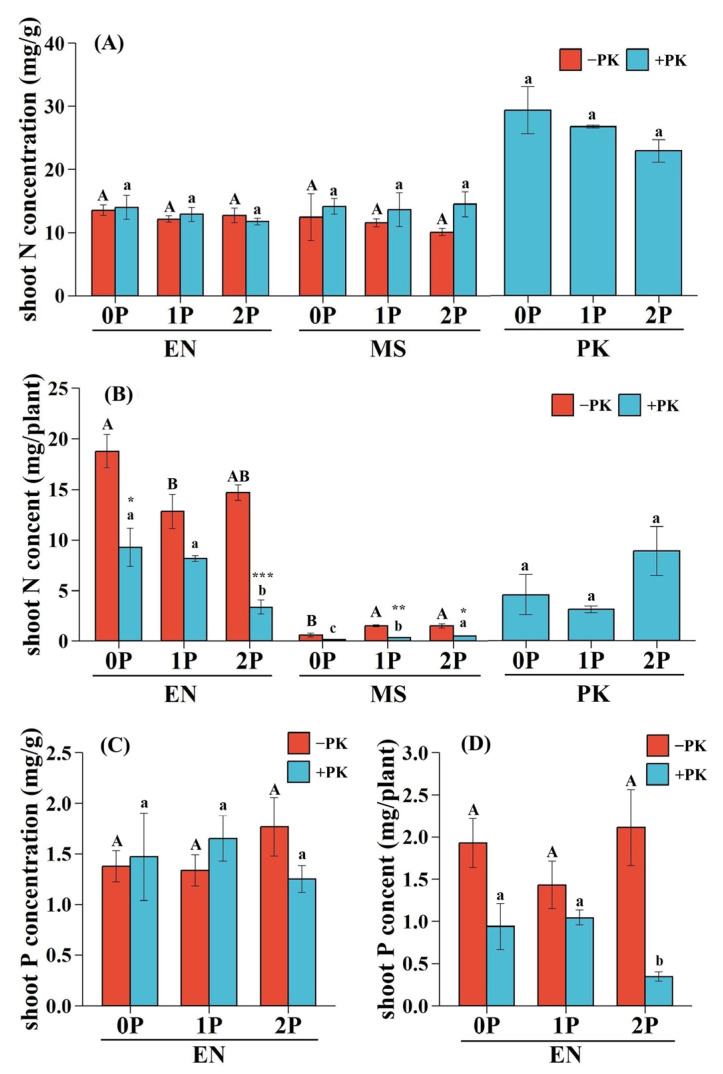
Shoot N and P status of hosts and hemiparasite in pots with or without *Pedicularis kansuensis* under different P conditions. (**A**) Shoot N concentration (mg/g of dry weight, mg/g); (**B**) shoot N concentration (mg per plant, mg/plant); (**C**) shoot P concentration (mg/g of dry weight, mg/g); (**D**) shoot P content (mg per plant, mg/plant). Data are mean ± 1 SE. Different uppercase letters indicate significant effects of P availability on host plants in pots without PK (−PK); different lowercase letters indicate significant effects of P availability on host and hemiparasite in pots with PK (+PK) (*p* < 0.05, LSD tests). Asterisks denote significant differences between treatments with or without PK under the same P level (* *p* < 0.05; ** *p* < 0.01; *** *p* < 0.001, two independent samples’ *t* test). Species: *Elymus nutans* (EN), *Medicago sativa* (MS), *P. kansuensis* (PK). P treatments: 0P, 0 mM/L Na_2_HPO_4_; 1P, 1.33 mM/L Na_2_HPO_4_; 2P, 2.66 mM/L Na_2_HPO_4_.

The effects of P availability and parasitism on N concentrations of *E. nutans* and *M. sativa*, as well as the P concentration of *E. nutans*, were analyzed. It was found that no significant single and interactive effects were observed (Table 2; Figure 4A,C). P availability and parasitism had single effects on the N contents of *E. nutans* and *M. sativa*, and parasitism (owing to larger η_P_^2^ values) played a more critical role in host N content than P levels (Table 2). The presence of *P. kansuensis* significantly reduced N contents in *E. nutans* and *M. sativa* (Figure 4B). Under low P conditions (0P and 1P), the N content of *M. sativa* was suppressed more severely than *E. nutans* (80.35% vs. 50.67% at 0P and 79.42% vs. 36.36% at 1P). However, under 2P, N suppression in *M. sativa* decreased (68.06%), while it intensified in *E. nutans* (77.35%). The increase in P availability significantly reduced the N content of *E. nutans*, particularly when parasitized by *P. kansuensis* (Figure 4B). On the other hand, the N contents of infected *M. sativa* and *P. kansuensis* increased with increasing P availability (Figure 4B).

According to the results of two-way ANOVA, parasitism had a significant effect on the P content of *E. nutans* (Table 2), while there was no notable decrease in the P content of *E. nutans* between treatments with or without *P. kansuensis* under the same P level (0P, 51.35%, *p* > 0.05; 1P, 27.12%, *p* > 0.05; 2P, 83.70%, *p* > 0.05; Figure 4D). In contrast, P availability did not have a noticeable effect on the P content of *E. nutans* (Table 2), but it significantly decreased with P increment when the grass host was infected by *P. kansuensis* (Figure 4D). The P contents of parasitized *M. sativa* and *P. kansuensis* could not be measured due to insufficient biomass.

## 3. Discussion

### 3.1. P Affected Host Selectivity of Root Hemiparasitic Plants Between Grass and Legume

To evaluate the host preference of *P. kansuensis* while controlling the “meeting chance” caused by differential root biomass, we compared the haustoria number per gram of dry weight between different host plants. The results showed a distinct foraging preference of *P. kansuensis* between grass and legume hosts, which was affected by P availability. Under 0P and 1P treatment, although *M. sativa* (legume) exhibited substantially lower root biomass than *E. nutans* (dominant grass) (Table 1), *P. kansuensis* developed significantly more haustoria on the legume host (Figure 1B), indicating that *P. kansuensis* preferred to forage legumes under low P conditions. Under high P conditions (2P), *P. kansuensis* decreased its preference for the legume host, shifting its parasitism towards the dominant *E. nutans* and forming more haustoria on its roots (Figure 1B). This study demonstrates that soil P availability influences the host selectivity of the root hemiparasitic plant *P. kansuensis* in a grass–legume co-culture.

The modulation of host selectivity by P in *P. kansuensis* altered their effects on the growth dynamics of grass and legume hosts, impacting their relationship in a grass–legume co-culture. When P levels were low, *M. sativa* was more strongly suppressed compared to *E. nutans*, whereas the opposite pattern was observed under high P conditions (Figure 2). These changes in host suppression suggest that hemiparasitic plants can alter interspecific competition in plant communities by affecting coexisting host species differently [37,38]. The suppression of dominant species like *E. nutans* by *P. kansuensis* under high P conditions (Figure 3) supports the hypothesis that parasitic plants reduce the competitive advantage of dominant species, facilitating coexistence and potentially increasing plant community diversity [1,17]. However, the overall productivity of the plant community was reduced in the presence of *P. kansuensis*, with the extent of suppression increasing at higher P levels (Figure 3). This suggests that the ecological benefits of hemiparasitic plants in promoting species coexistence may come at the cost of reduced ecosystem productivity.

### 3.2. Potential Mechanisms of P Affect Host Selectivity in Root Hemiparasitic Plants

Several studies have shown that root hemiparasitic plants can distinguish legumes from grasses [8], and they generally exhibit stronger suppression on legumes in a multispecies system [9,39]. However, the mechanisms behind this phenomenon still need to be further investigated. Our study confirmed the ability of root hemiparasitic plants to differentiate between legumes and grasses and also suggested that their host selectivity may vary with changes in P availability.

The P-mediated regulation of host–parasite interactions was initially established in a single host system, where high levels of P inhibited haustorium formation in *Rhinanthus minor* on *Lolium perenne* by causing root thickening that mechanically blocked haustoria penetration [40]. In our two-host experiment, despite not quantitatively assessing the root morphological parameters and haustoria structure, both hosts exhibited significantly suppressed growth performance (shoot DW, *E. nutans* 78.68%, *M. sativa* 64.41%; root DW, *E. nutans* 45.54%, *M. sativa*: 36.65%) and nutrient status (shoot N content, *E. nutans* 77.35%, *M. sativa* 68.06%) under high P conditions, indicating their susceptibility to parasitism by *P. kansuensis*. Therefore, the changes in the capacity of *P. kansuensis* to develop haustoria on different hosts under various P levels imply that the P-mediated host selection of the root hemiparasite operates through alternative mechanisms rather than purely physical barriers.

Recent studies show that strigolactones (SLs), such as orobanchol and 5-deoxystrigol, act as chemoattractants for host tropism (the initial step of host selectivity) in both the facultative root hemiparasite *Phtheirospermum japonicum* and the obligate root hemiparasite *Striga hermonthica* [32]. This suggests that host preference in root hemiparasitic plants is likely driven by host-derived chemical signals, whose production is influenced by P availability in growth media. In this study, P availability affected haustoria formation on legume roots (Figure 1A), implying that host-derived factors, like flavonoids, phenolics, or quinones [32,41], may also contribute to host selectivity. Since P deficiency elevates legume secretion of SLs [31], flavonoids [42,43,44], and phenolic acids [45], altered production of these signals likely drives P-dependent host preference shifts. This explains the preference of *P. kansuensis* to *M. sativa* under low P (likely due to elevated chemoattractants) but to grasses under high P conditions (where reduced legume signals enable grass dominance). Identifying specific chemical signals and their nutrient-regulated mechanisms is essential to understanding host selectivity in root hemiparasitic plants.

### 3.3. Impact of Soil P Availability on Growth Performance of Hemiparasites

The growth of *P. kansuensis* was positively influenced by high P availability, as evidenced by increased shoot and root biomass under 2P conditions (Table 1). These findings are consistent with previous studies showing that nutrient addition enhances hemiparasite growth [24,46], either through improved host resource availability [47] or direct nutrient uptake by hemiparasites [48]. While we did not quantify the nutrient transferred from host to hemiparasite or evaluate the photosynthesis capability of the root hemiparasite in this study, we found that the root–shoot ratio of *P. kansuensis* was higher in the 2P treatment than in the 1P treatment (Table 1), implying that root hemiparasitic plants invest more energy in root growth and improve their nutrient absorption capability in high P conditions.

On the other hand, some studies suggested that fertilization had a negative effect on the growth of root hemiparasitic plants because nutrient addition facilitated the growth of host plants, leading to increased competition from host plants for light [4,49]. For example, Liu et al. [35] found that adding P fertilizer suppressed the above-ground biomass of *P. kansuensis* in a field study, as the host plants outcompeted the hemiparasite under high nutrient conditions. The discrepancy in the effects of P on the growth of *P. kansuensis* between our study and Liu’s research may be partly explained by differences in plant growth conditions, as various factors such as nutrients, water, and soil microbes can influence the interaction between host and hemiparasitic plant [21,50].

Interestingly, the growth of *P. kansuensis* under P-deficient conditions (0P) was not significantly lower than 1P treatment (Table 1). This resilience can be attributed to its parasitic strategy, which allows it to obtain critical nutrients from host plants. This capacity to thrive in nutrient-poor environments likely explains the prevalence of root hemiparasitic plants in habitats with low soil fertility [48].

### 3.4. Implications for Management of Parasitic Plants

This study highlights the importance of soil P availability in influencing the dynamics of parasitic plant–host interactions. Manipulating soil nutrient levels, particularly P availability, could be a potential strategy for managing the impacts of parasitic plants like *P. kansuensis* on agricultural systems and natural plant communities. For example, in systems where legumes are the preferred hosts, increasing P availability could shift parasitism toward grasses, potentially reducing damage to economically important legume crops. Future research should focus on field-based experiments to validate the findings of this study and explore the broader ecological and practical implications of nutrient-mediated host selectivity in root hemiparasitic plants. Additionally, integrating chemical analyses of host root exudates could provide deeper insights into the mechanisms driving host preference and its modulation by soil nutrients.

It is worth noting that the cultivation medium in this study was sterilized to eliminate interference from soil microorganisms on the host selectivity of root hemiparasitic plants. However, in natural soil environments, soil microorganisms, particularly arbuscular mycorrhizal fungi (AMF), are ubiquitous [51]. AMF generally form symbiotic relationships with grasses and legumes, influencing interactions between plant groups and being affected by soil P levels [52,53,54]. Therefore, future research should investigate the role of soil microorganisms, such as AMF and potentially other types, in mediating interactions between parasitic plants and a wider range of potential host plants under varying P availability, employing mixed-plant designs similar to the one used here.

## 4. Materials and Methods

### 4.1. Experimental Design

*Elymus nutans* (grass) and *Medicago sativa* (legume) were selected as host species due to their known preference by *P. kansuensis* (PK) and their common use in alpine grassland restoration efforts in China. A factorial pot experiment was conducted based on a grass–legume co-culture design, with the presence/absence of *P. kansuensis* (+PK and −PK) and three levels of P availability (0 mM/L Na_2_HPO_4_ [0P], 1.33 mM/L Na_2_HPO_4_ [1P], and 2.66 mM/L Na_2_HPO_4_ [2P]), based on the Long Ashton nutrient solution formula [20,55]. Each treatment combination was replicated with 6 pots for +PK (one grass, one legume, and one parasite) and 5 pots for −PK (one grass and one legume), resulting in a total of 33 pots.

### 4.2. Plant Materials

Seeds of *P. kansuensis* and *E. nutans* were collected from Bayanbulak Grassland (83°42′ E, 42°52′ N, 2472 m) in Xinjiang Uygur Autonomous Region, China, in late August 2016. *M. sativa* seeds were sourced commercially in 2010. All seeds were stored at 4 °C until use. Seeds were surface-sterilized with 4.5% sodium hypochlorite for 5 min and rinsed 5 times with distilled water. Seeds were germinated on moistened filter paper in Petri dishes and incubated at 18/25 °C (day/night) with a 12 h photoperiod and a light intensity of 22.2 μmol photons m^−2^s^−1^.

### 4.3. Planting and Growth Conditions

Based on our grass–legume co-culture design, germinated *E. nutans* and *M. sativa* seedlings were planted in pairs in the center of each pot, spaced 2 cm apart. After one week, a single germinated *P. kansuensis* seedling was added to +PK pots, forming an equilateral triangle with the host seedlings. Each pot contained 3.2 kg of growth substrate, a mixture of 10% soil material from Kunming Botanical Garden and 90% river sand. The soil and sand were sieved through a 1 mm sieve before use. Available N, P, and K concentrations of the growth substrate were around 14.3, 2.7, and 62.4 mg kg^−1^ dry substrate. To eliminate the influence of soil microbes on the host selection of root hemiparasitic plants, we utilized autoclaved substrate (121 °C for 2 h).

Pots were maintained in a greenhouse at Kunming Institute of Botany, Chinese Academy of Sciences, under natural light conditions with temperatures ranging from 15 to 35 °C. The experiment ran from 23 June to 30 October 2017, 129 days. P fertilizer solutions were applied weekly (20 mL per pot) two weeks after planting, corresponding to the specified P levels for each treatment. Pots were randomized weekly to minimize positional effects.

### 4.4. Harvest and Sampling

Only pots where all plants survived until the end of the experiment were included in the analysis (three pots under 0P conditions with PK, five pots under 0P and 1P conditions without PK, four pots under other conditions). At harvest, shoots and roots were separated. The roots of each plant species were identified morphologically and separated before being stored in 50% alcohol for later measurement. Three replicates for each +PK treatment were randomly selected to analyze the number of haustoria (HN) produced by *P. kansuensis*. Haustoria were categorized into two types: (1) presumably functional haustoria (PFH) that attach to host roots with distinct xylem bridges [56], and (2) non-functional haustoria (NFH) without distinct xylem bridges. The number of PFH attached to each host was counted separately. The total number of haustoria produced by *P. kansuensis* was the sum of the number of PFH attached to each host and the number of NFH. The incidence of haustorium formation was recorded as the number of haustoria per gram of dry root samples [57]. Plant shoot and root dry weights (DWs) were determined after oven drying at 75 °C for 48 h.

### 4.5. Measurement of Shoot N and P Concentrations

For nutrient analysis, shoot N and P concentrations were measured from the three replicates per treatment that were used for examining haustoria numbers. N concentration was determined using an elemental analyzer (Vario Macro Cube, Elementar, Germany), while P concentration was measured with the phosphovanado-molybdate method on a spectrophotometer (L6S, Shanghai Yidian Analytical Instrument Co., Ltd., Shanghai, China). Due to limited biomass in certain pots, shoot P concentration for *M. sativa* and *P. kansuensis* was not analyzed. Shoot N or P contents were calculated by multiplying the element concentration and shoot DW.

### 4.6. Statistical Analyses

Two-way ANOVA was used to evaluate the effects of P availability and parasitism on shoot dry weight (DW), root DW, shoot N, and P status (concentration and content) of *E. nutans* and *M. sativa*, as well as total shoot biomass per pot. One-way ANOVA followed by the LSD test assessed the influence of P availability on growth performance, nutrient status, and haustorium formation. Independent sample *t*-tests were used to analyze the effects of parasitism on host growth and nutrient status, as well as differences in haustorium formation by *P. kansuensis* between host species. Before analysis, data were assessed for normality and homogeneity. For specific datasets, data were transformed by log10 to meet assumptions of normality and homogeneity of variances. Mann–Whitney U tests were employed for datasets with non-normal distributions. Statistical analyses and visualization were conducted in R (version 4.4.2) and R-Studio using packages dplyr [58], emmeans [59], effectsize [60], car [61], ggpubr [62], and ggplot2 [63].

## 5. Conclusions

This study demonstrates that host selectivity of the root hemiparasitic plant *P. kansuensis* is influenced by soil P availability. Under low P conditions, *P. kansuensis* preferentially parasitized the legume host *M. sativa*, forming more haustoria and causing significant biomass reduction in the legume host despite much higher grass biomass in the host mixture. In contrast, under higher P levels, *P. kansuensis* shifted its parasitism toward the grass host *E. nutans*, leading to more severe suppression of grass growth. These findings emphasize the significance of soil P status in shaping the interactions between parasitic plants and their hosts. They also have practical implications for mitigating the damage caused by parasitic weeds in agricultural and natural ecosystems via adjusting nutrient supply. Future research should explore these interactions in field settings and investigate the chemical and physiological mechanisms underlying host preference. Such studies will deepen our understanding of parasitic plant ecology and help develop strategies for managing their impact on plant communities.

## Figures and Tables

**Table 1:** Comparisons of growth performance of *Elymus nutans*, *Medicago sativa*, and *Pedicularis kansuensis* under different parasitism or P conditions.

Species	P Level	Shoot Dry Weight (g)		Root Dry Weight (g)		Root–Shoot Ratio	
		−PK	+PK	−PK	+PK	−PK	+PK
***E. nutans***	0P	1.35 ± 0.07 Aa	0.66 ± 0.11 Ab	0.89 ± 0.06 Aa	0.46 ± 0.09 Ab	0.66 ± 0.04 Aa	0.73 ± 0.16 Aa
	1P	1.13 ± 0.11 Aa	0.56 ± 0.08 ABb	0.71 ± 0.08 Aa	0.49 ± 0.08 Aa	0.62 ± 0.02 Aa	0.88 ± 0.06 Aa
	2P	1.25 ± 0.08 Aa	0.26 ± 0.04 Bb	0.74 ± 0.06 Aa	0.39 ± 0.12 Aa	0.60 ± 0.05 Ab	1.46 ± 0.42 Aa
***M. sativa***	0P	0.05 ± 0.01 Ba	0.01 ± 0.00 Bb	0.05 ± 0.01 Ba	0.01 ± 0.00 Bb	1.02 ± 0.08 Aa	1.26 ± 0.58 Aa
	1P	0.12 ± 0.02 Aa	0.02 ± 0.00 Bb	0.10 ± 0.01 Aa	0.02 ± 0.00 Bb	0.89 ± 0.10 ABa	0.77 ± 0.08 Aa
	2P	0.12 ± 0.02 Aa	0.04 ± 0.01 Ab	0.08 ± 0.01 ABa	0.05 ± 0.01 Aa	0.72 ± 0.05 Bb	1.31 ± 0.12 Aa
***P. kansuensis***	0P	--	0.15 ± 0.07 AB	--	0.03 ± 0.01 B	--	0.18 ± 0.02 B
	1P	--	0.12 ± 0.01 B	--	0.02 ± 0.00 B	--	0.17 ± 0.02 B
	2P	--	0.35 ± 0.08 A	--	0.13 ± 0.02 A	--	0.38 ± 0.07 A

Data are presented as mean ± 1 SE. Different uppercase letters in the same column indicate a statistically significant difference at *p* < 0.05 levels between three P availability based on one-way ANOVA followed by LSD post hoc tests; different lowercase letters in the same row indicate a statistically significant difference at *p* < 0.05 levels between −PK (without parasite) and +PK (with parasite) treatment based on two independent samples’ *t* test. -- indicates data for *P. kansuensis* was not collected in the -PK treatment due to their absence.

**Table 2:** Two-way ANOVA results (F and η_P_^2^ values) for the effects of phosphorus availability (P) and parasitism (Pa) on shoot N and P status of *Elymus nutans* (EN) and *Medicago sativa* (MS).

		N Concentration				P Concentration			N Content				P Content		
		EN		MS		EN		MS	EN		MS †		EN		MS
	df	F	η_P_^2^	F	η_P_^2^	F	η_P_^2^		F	η_P_^2^	F	η_P_^2^	F	η_P_^2^	
P	2, 12	1.091	0.15	0.117	0.02	0.063	0.01	**--**	7.717 **	0.56	30.153 ***	0.83	0.358	0.06	--
Pa	1, 12	0.010	0.00	2.484	0.17	0.028	0.00	**--**	63.166 **	0.84	101.813 ***	0.89	21.840 **	0.65	--
P * Pa	2, 12	0.334	0.05	0.239	0.04	1.440	0.19	**--**	3.479	0.37	1.057	0.15	3.169	0.35	--

Values suggesting significant effects were given in bold (** *p* < 0.01; *** *p* < 0.001). – indicates that data were unanalyzed because samples in some pots were insufficient for P measurement. † indicates that data were log 10 transformed before analyses. -- indicates that data could not be collected and analyzed because of insufficient biomass from parasitized *M. sativa* and *Pedicularis kansuensis* plants.

## Data Availability

The original contributions presented in the study are included in the article; further inquiries can be directed to the first author.
